# *TTN* truncation variants produce sarcomere-integrating proteins of uncertain functional significance

**DOI:** 10.1172/JCI175206

**Published:** 2024-01-16

**Authors:** J. Travis Hinson, Stuart G. Campbell

**Affiliations:** 1 The Jackson Laboratory for Genomic Medicine, Farmington, Connecticut, USA.; 2 Cardiology Center, UConn Health, Farmington, Connecticut, USA.; 3 Department of Biomedical Engineering, Yale University, New Haven, Connecticut, USA.; 4 Department of Cellular and Molecular Physiology, Yale School of Medicine, New Haven, Connecticut, USA.

## Abstract

Titin (TTN) is one of the largest and most complex proteins expressed in humans, and truncation variants are the most prevalent genetic lesion identified in individuals with dilated cardiomyopathy (DCM) or other disorders of impaired cardiac contractility. Two reports in this issue of the *JCI* shed light on a potential mechanism involving truncated TTN sarcomere integration and the potential for disruption of sarcomere structural integrity. Kellermayer, Tordai, and colleagues confirmed the presence of truncated TTN protein in human DCM samples. McAfee and authors developed a patient-specific TTN antibody to study truncated TTN subcellular localization and to explore its functional consequences. A “poison peptide” mechanism emerges that inspires alternative therapeutic approaches while opening new lines for inquiry, such as the role of haploinsufficiency of full-length TTN protein, mechanisms explaining sarcomere dysfunction, and explanations for variable penetrance.

## TTN structure and function

The sarcomere is the fundamental contractile unit of the myocyte and is commonly subdivided into Z-disc, I-band, A-band, and M-line regions, and the titin (TTN) protein spans half the sarcomere. The *TTN* gene has 364 exons (meta-transcript, Ensembl ID: ENST00000589042), which are differentially spliced in heart and skeletal muscle through development and disease (1). *TTN* cardiac expression is regulated by two promoters: a major promoter that regulates the expression of N2BA, N2B, and novex isoforms (novex-1, novex-2, and novex-3), and an internal promoter located at the I-/A-band junction that regulates the expression of Cronos, a developmentally regulated TTN isoform that is missing Z-disk and most I-band exons (2). N2BA is the longest isoform, ranging in size from approximately 3.3 to 3.8 MDa, and is the predominant isoform in the developing heart, while N2B is approximately 3 MDa and lacks many I-band exons, including those encoding PEVK (enriched with proline, glutamate, valine, and lysine residues), and other extensible segments. In heart failure, the stoichiometry of N2BA to N2B shifts higher to favor the more extensible N2BA isoform (3) that resembles the ratio identified in fetal hearts (4). TTN is required for sarcomere assembly and twitch contraction (5) through interactions with multiple partners including α-actinin (6) at the Z-disc and obscurin (7) at the M-line. Contributing to passive force, TTN’s distensible, spring-like I-band and PEVK domains can be modified by phosphorylation (8) and other factors. Moreover, TTN functions as a mechanotransduction signaling hub with stretch-dependent TTN-protein interactions (9). TTN’s complex regulation and functions as well as its enormous size have limited our understanding of its dysfunction in cardiac disorders until recently.

## *TTN* variants in health and disease

Heterozygous *TTN* truncation variants (*TTN*tvs) are the most prevalent genetic lesion identified in dilated cardiomyopathy (DCM), a disorder associated with cardiac chamber enlargement and impaired contractile function (10). DCM prevalence has been estimated at approximately 1 in 200 individuals, and *TTN*tvs can be identified in up to 25% of individuals with DCM (11). DCM risk may depend on *TTN*tv localization, as *TTN*tvs localized to exons encoding A-band residues have a higher pathogenicity compared with those localized in differentially spliced exons such as those encoding I-band residues (12). DCM risk is elevated up to approximately 50-fold in carriers of *TTN*tvs, but healthy individuals may also harbor *TTN*tvs, and explanations for this variable penetrance are incomplete but probably related to genetic background. Acquired risk factors are yet to be determined. *TTN*tvs are also reported in peripartum cardiomyopathy (13) and cardiomyopathy associated with chronic alcohol consumption, suggesting that additional stressors act in concert with *TTN*tvs (14).

## The sarcomere integrates *TTN*tv-generated truncated TTN protein

**Figure 1:**
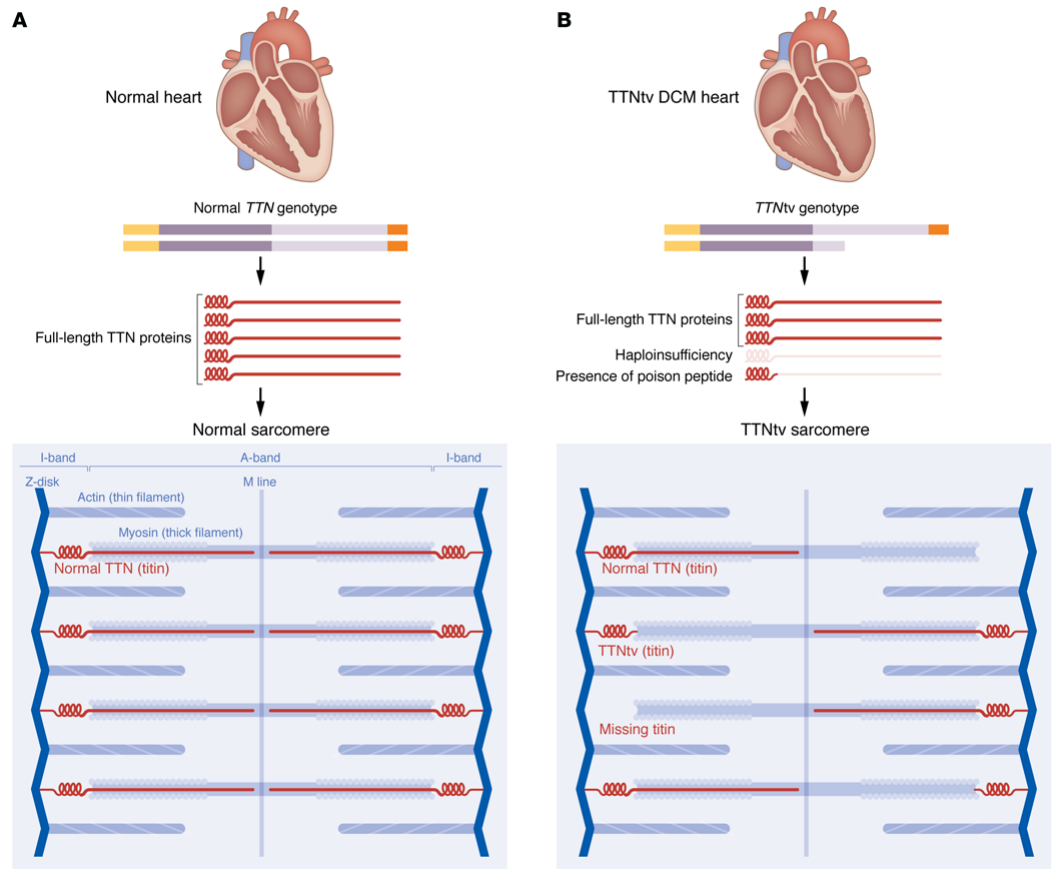
A dominant-negative or poison peptide model accounts for how some *TTN*tvs may contribute to DCM pathogenesis. Heterozygous *TTN*tvs are the most prevalent genetic lesion identified in DCM, but the disease mechanism remains elusive. Accumulating evidence shows that some TTNtvs integrate within the sarcomere and are load bearing. At the same time, TTNtvs are also shown to reduce the amount of full-length TTN protein (haploinsufficiency). At present, either mechanism remains a plausible driver of DCM, with the possibility that both contribute in tandem.

Recent studies, including two presented in this issue of the *JCI*, have utilized vertical agarose gel electrophoresis (VAGE) to evaluate expression of the *TTN*tv allele in human DCM myocardium and model systems. An early study, relying on linkage analysis of the *TTN* locus on chromosome 2q31, used VAGE to evaluate human myocardial lysates with an A-band TTNtv (c.43628insAT) (15). The presence of truncated TTN protein of the c.43628insAT allele was further corroborated by the same group in a functional study characterizing a c.43628insAT knockin mouse model (5). Indeed, the truncated TTN protein was estimated to be approximately 1% of full-length TTN. Additional studies of two TTNtv DCM cohorts also validated the presence of low-level truncated TTN species from human myocardial specimens, largely corroborating earlier work from human cardiomyocytes differentiated in vitro from an induced pluripotent stem cell (iPSC) model derived from an individual with DCM who was a *TTN*tv carrier (16, 17). Now in the *JCI*, Kellermayer, Tordai, and co-authors (18) confirmed the presence of truncated TTN protein in additional human DCM samples, while further supporting previous studies that had demonstrated truncated TTN protein within myofibril fractions isolated by biochemical approaches (16) or by colocalization microscopy (19). In the *JCI* study, Kellermayer, Tordai, and colleagues question the role of *TTN* haploinsufficiency (18). However, the findings support a dominant-negative or poison peptide genetic mechanism for some *TTN*tvs (Figure 1).

To begin studying the functional impact of TTNtvs, McAfee et al. now report an elegant study (20). The authors developed a patient-specific, custom TTNtv antibody that was designed to specifically recognize the 32 amino acid neoepitope encoded by a DCM-associated heterozygous exon 329 frameshift mutation corresponding to the A-band structural domain (termed TTNtvA). Since the custom antibody did not bind to WT TTN protein, this tool could be used to study truncated TTN subcellular localization and to explore its functional consequences. Despite the lack of an M-line domain, TTNtvA protein was identified in skinned human cardiomyocyte fragments in the sarcomere thick filament/A-band region. This location was predicted for TTNtvA, given the position of its termination codon within the mid A-band region. To further examine the functional properties of TTNtvA protein, the research group stretched cardiomyocyte fragments from short to supraphysiological sarcomere lengths and imaged TTNtvA using the custom antibody recognizing its C-terminus. If TTNtvA were to maintain its terminal A-band positioning after stretching, and not recoil to either the Z-disc or other subsarcomere region, it could be reasonably inferred that TTNtvA could bear load across the sarcomere. Indeed, McAfee and authors observed no change in TTNtvA positioning with stretch unless potassium chloride, a thick filament disruptor, was added (20). These results demonstrate how truncated TTN can integrate into the sarcomere and bear load in a human myocardial sample. While this finding was a step forward, the functional consequences of a load-bearing, truncated TTN remain completely unknown.

To consider how truncated TTN protein impacts sarcomere structure and function, Kellermayer, Tordai, and colleagues (18) also report on an analysis of human TTNtv myocardial samples using super-resolution stimulated emission depletion (STED) microscopy with TTN antibodies recognizing different epitopes either common or exclusive to full-length TTN or truncated TTN. As in the study by McAfee et al. (20), imaging was performed in conjunction with mechanical stretch. In brief, Kellermayer, Tordai, and co-authors observed that truncated TTN was expressed in myofibril fractions and that mechanical stretch elicited reduced A-band extensibility and increased distance between the titin kinase domain and the M-line, suggesting putative functional consequences of TTNtvs. Their report of structural and functional consequences may need to be further validated using reagents that specifically recognize truncated TTN proteins, but it nonetheless supports a poison peptide mechanism (18).

## TTNtvs also reduce full-length TTN protein levels

In addition to truncated TTN production from *TTN*tv alleles, *TTN*tvs have also been reported to lead to reduced full-length TTN protein levels in human myocardial samples, suggesting a haploinsufficiency genetic mechanism (Figure 1). Defined as the inability of the single WT *TTN* allele to produce sufficient full-length TTN protein to maintain normal cardiac function*,* in two recent studies (16, 17) and in the report by Kellermayer, Tordai, and colleagues (18), *TTN*tv DCM myocardial samples expressed approximately 15% less full-length TTN protein relative to other DCM or control samples. Similar results were observed in human iPSC–derived cardiomyocyte models composed of similar TTNtvs, although with greater reductions of approximately 50% relative to controls (19). The role of reduced TTN protein levels is less well understood in DCM pathogenesis, but potential mechanisms gleaned from functional studies implicate impaired sarcomere function (19, 21, 22) and cell-signaling pathways (21).

## Questions and future directions

Secondary to TTN’s large size and complex structure, it has been a challenge to the field to delineate the functional consequences of TTNtvs. The McAfee et al. study (20), with its unique strategy for exclusive detection of truncated TTN, clarifies the presence and behavior of TTN within the sarcomere. However, future studies will be essential to understand how this protein, despite a capacity for bearing mechanical load, differs from full-length TTN. Specifically, does TTNtv’s lack of thick filament–encoding residues impair force production and disturb other protein interactions such as M-line interactions, or could it disturb cardiac function through activation of the unfolded protein response as recently reported by others (17)? While McAfee et al. (20) provide some insights into localization and load capacity, more work is needed to fully understand the functionality of truncated TTNs and whether they differ for distinct truncations. Similarly, Kellermayer, Tordai, and co-authors (18) report structural alterations in DCM samples from individuals carrying TTNtvs, but made several assumptions based on indirect studies from heterogenous samples obtained from explanted human tissue. In considering all the studies together, it appears that the combination of reduced total full-length TTN and the insertion of a truncated peptide into the sarcomere is present and likely plays a role in disease pathogenesis.

Models and approaches are needed to experimentally dissect the functional consequences of sarcomere-integrating TTNtv proteins as well as full-length TTN protein haploinsufficiency. One such approach may be to combine human iPSC models and genome-editing technologies such as CRISPR. Despite maturation limitations, human iPSC–derived cardiomyocytes can be developed using CRISPR genetic ablation of truncated TTN protein to explore the specific functional impact of these poison peptides. Through the use of a 3D cardiac microtissue model to study sarcomere contractile function in a biomimetic context, truncated TTN ablation was shown to partially rescue sarcomere function, thus implicating truncated TTN as a sarcomere poison. Because the rescue was partial, this study also supported a combinatorial genetic mechanism including haploinsufficiency (19). Continued efforts toward a precise understanding of how TTNtvs lead to DCM and other cardiomyopathies will catalyze the development of mechanistically precise therapies targeting one of the most important heart failure causes in the field.

## Version 1 01/16/2024

Electronic publication
